# Advanced MRI features in relapsing multiple sclerosis patients with and without CSF oligoclonal IgG bands

**DOI:** 10.1038/s41598-020-70693-9

**Published:** 2020-08-13

**Authors:** Lin Zhao, Jill Abrigo, Qianyun Chen, Cheryl Au, Angel Ng, Ping Fan, Vincent Mok, Wei Qiu, Allan G. Kermode, Alexander Y. Lau

**Affiliations:** 1 Department of Medicine and Therapeutics, Prince of Wales Hospital, Chinese University of Hong Kong, Hong Kong SAR, China; 2 Department of Imaging and Interventional Radiology, Prince of Wales Hospital, Chinese University of Hong Kong, Hong Kong SAR, China; 3 Neurology Department, Third Affiliated Hospital, Sun Yat-Sen University, Guangzhou, China; 4 Centre for Neuromuscular and Neurological Disorders, Perron Institute, University of Western Australia, Perth, Australia

**Keywords:** Neuroscience, Diseases of the nervous system, Multiple sclerosis

## Abstract

Oligoclonal IgG bands (OCB) in cerebrospinal fluid (CSF) are important in diagnosis of multiple sclerosis (MS). We evaluated the MRI features of clinically definite MS subjects with and without CSF-OCB. Relapsing MS subjects were recruited from a prospective registry in a university center. CSF-OCB were detected using isoelectric focusing and lgG-specific immunofixation. MRI metrics including brain volumes, lesion volumes and microstructural measures, were analyzed by FMRIB Software Library (FSL) and Statistical Parametric Mapping (SPM)*.* Seventy-five subjects with relapsing MS were analyzed. Forty-four (59%) subjects had an interval MRI at around 1 year. CSF-OCB were detected in 46 (61%) subjects. The OCB-positive group had a higher proportion of cerebellar lesions than the OCB-negative group (23.9% vs. 3.4%, *p* = 0.057). Except for amygdala volumes which were lower in the OCB-positive group (*p* = 0.034), other regional brain volumes including the subcortical deep gray matter and corpus callosum were similar. The two groups also showed comparable brain atrophy rate. For DTI, the OCB-positive group showed significantly higher mean diffusivity (MD) value in perilesional normal-appearing white matter (*p* = 0.043). Relapsing MS patients with and without CSF-OCB shared similar MRI features regarding volumetric analyses and DTI microstructural integrity.

## Introduction

Oligoclonal IgG bands (OCB) in the cerebrospinal fluid (CSF) are one of the most sensitive biomarkers in the diagnostic work-up of multiple sclerosis (MS), and inclusion of their presence into the updated McDonald criteria has improved clinical diagnosis^1^. Up to 95% of Caucasian patients and 60% of Asian patients with MS are positive for CSF-OCB^2,3^. Limited data support that CSF-OCB may be involved in the pathogenesis of MS; some pathological studies revealed that tissue damage may be underpinned by other types of oligoclonal bands such as IgM^4^. Meanwhile, the relationship between CSF-OCB status and disease characteristics, such as clinical course, disease progression, responses to disease modifying therapies, remain unclear^3,5^.

Magnetic resonance imaging (MRI) is now standard of care in the management of MS, allowing diagnosis and monitoring of treatment response in clinical practice^6^. Compared to conventional MRI alone, the use of high-resolution images, advanced pulse sequences and advanced computational techniques provides a more comprehensive information on the pathophysiological extent of disease. For instance, volumetric imaging allows a more precise quantitative assessment of brain atrophy which in turn has been shown to occur early in the disease course and is associated with disease progression of MS^7,8^. Recent clinical trials have incorporated brain atrophy, at an annual brain volume loss (BVL) rate threshold of 0.4%, as a secondary outcome measure and marker of neurodegeneration in MS^9^. Meanwhile, diffusion tensor imaging (DTI) allows microstructural assessment of white matter integrity by quantifying the magnitude and directionality of the molecular diffusion of water along axonal tracts. Diffusion in normal myelinated axons tends to be anisotropic and parallel to tracts; in contrast, damaged white matter will return lower fractional anisotropy (FA) and higher mean diffusivity (MD). Such changes in these DTI metrics reflect histologic injury but may also shed light on mechanisms responsible for irreversible disability accumulation^10^. Complementary voxel-wise analysis using tract-based spatial statistics (TBSS) allows further investigation of damage in specific tracts in the cerebellar peduncles and corpus callosum which are related to disability progression in MS^11,12^.

The association between CSF-OCB status and MRI features remains unclear. Previous studies on the MRI differences between OCB-positive and OCB-negative patients have been limited to traditional MRI parameters such as anatomical lesion locations or visually graded brain atrophy, and results have been inconsistent^13,14^. Thus, in this study, we explored the association between CSF-OCB status and advanced MRI metrics that are related to disease prognosis. Given the variability of MS phenotypes, we focused on relapsing–remitting multiple sclerosis (RRMS) patients.

## Results

### Participants and clinical features

Seventy-five patients with RRMS were included in this study. All subjects had volumetric T1 and FLAIR brain scans; spinal cord MRI scans were available for 72 patients (95%) and DTI scans were successfully acquired for 67 (89%) patients. Follow-up MRI scans and EDSS assessment were acquired for 44 (59%) patients. The baseline demographics and clinical characteristics are shown in Table 1. The overall CSF-OCB positivity rate was 61% (46 patients). Patients who were positive for CSF-OCB had longer disease duration (4.84 vs. 3.26 years, *p* = 0.025) than patients in the OCB-negative group. There were no significant differences between the two groups in terms of sex, age at disease onset, annual relapse rate (ARR) before baseline, Expanded Disability Status Scale (EDSS) scores and disease modifying treatments (DMT) usage at baseline.

**Table 1:** Baseline demographics and clinical characteristics of all RRMS patients according to CSF-OCB status.

	ALL (n = 75)	OCB-negative (n = 29)	OCB-positive (n = 46)	*p*-value
Females, n (%)	58 (77.3%)	25 (86.2%)	33 (71.7%)	0.15^a^
Age at disease onset, years, mean (SD)	28.94 (8.02)	30.14 (8.94)	28.17 (7.39)	0.30^b^
Disease duration, years, median (IQR)	4.39 (1.62–7.62)	3.26 (1.30–5.34)	4.84 (2.22–9.86)	**0.025**^**c**^
Baseline ARR, median (IQR)	0.30 (0.08–0.77)	0.24 (0.06–0.78)	0.30 (0.08–0.76)	0.99^c^
EDSS at baseline, median (IQR)	0.0 (0.0–2.0)	0.0 (0.0–1.75)	0.0 (0.0–2.0)	0.77^c^
DMT use, n (%)	62 (82.7%)	24 (82.8%)	38 (82.6%)	0.99^a^

Significant *p*-values are shown in bold.

*RRMS* relapsing–remitting multiple sclerosis, *CSF* cerebrospinal fluid, *OCB* oligoclonal IgG bands, *ARR* annual relapse rate, *EDSS* Expanded Disability Status Scale, *DMT* disease modifying therapy, *SD* standard deviation, *IQR* interquartile range.

The difference of the means or ranks was calculated using ^a^Pearson chi-square test, ^b^the Student t test or ^c^Mann-Whitney rank sum test.

### MRI features

**Figure 1:**
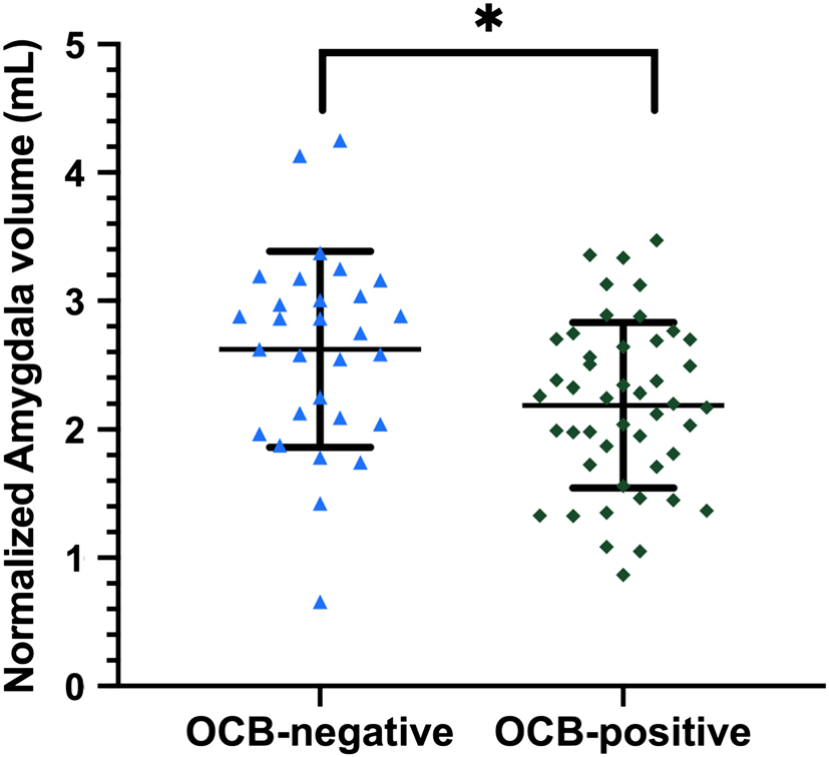
Difference in normalized amygdala volumes between OCB-negative group and OCB-positive group. *OCB* oligoclonal IgG bands, *p < 0.05. The horizontal lines were at mean with standard deviation. ANCOVA test with disease duration as the covariate was applied. The *p*-value was adjusted for multiple comparisons using the false discovery rate method.

**Figure 2:**
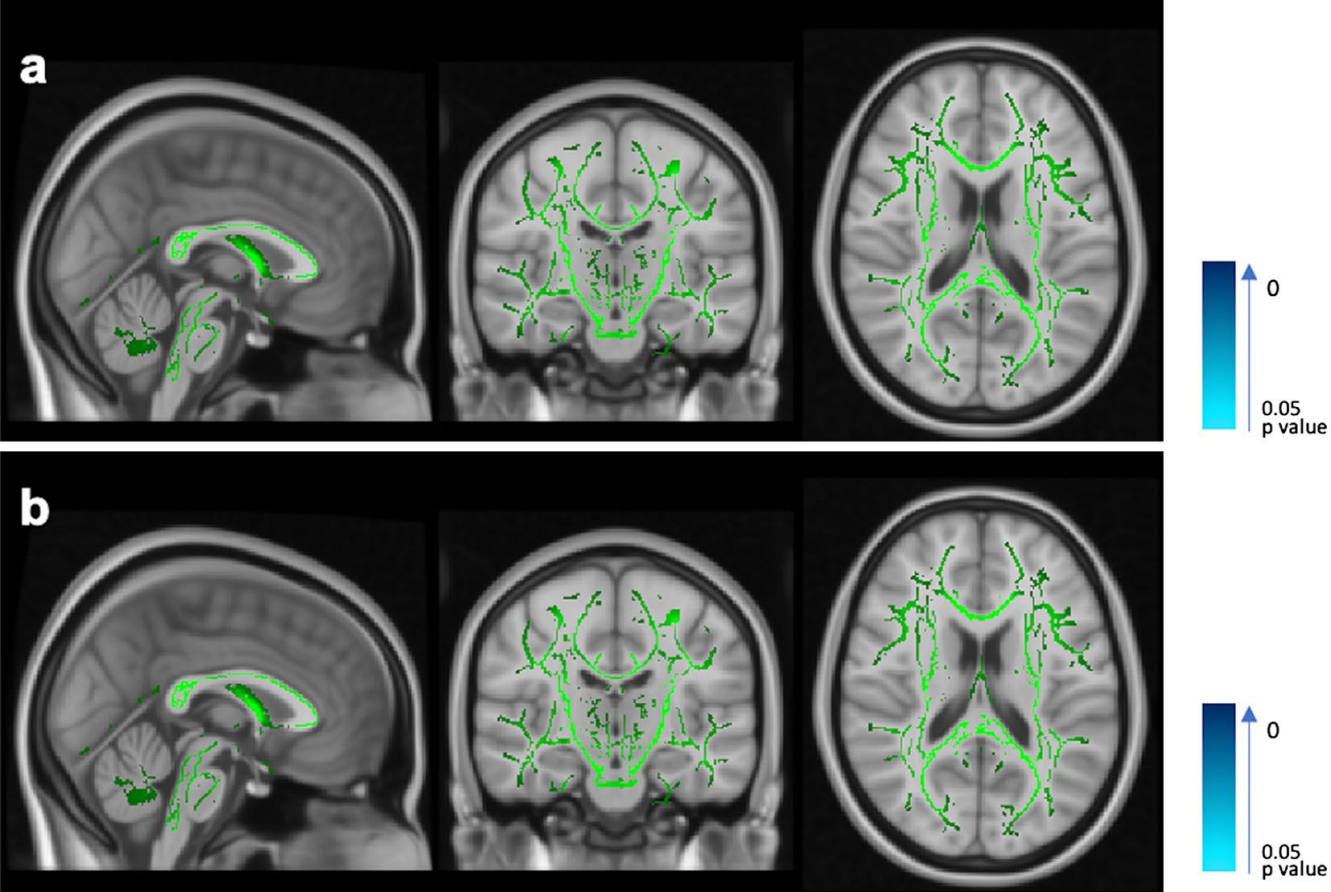
Tract-based spatial statistics (TBSS) showing mean fractional anisotropy (FA) and mean diffusivity (MD) skeletons in MNI 1 mm standard space. Tracts in green show no significant difference, whereas tracts in blue show significant difference. No significant difference of FA values (**a**) and MD values (**b**) were found on white matter tracts between patients with oligoclonal IgG bands (OCB) and without OCB.

**Figure 3:**
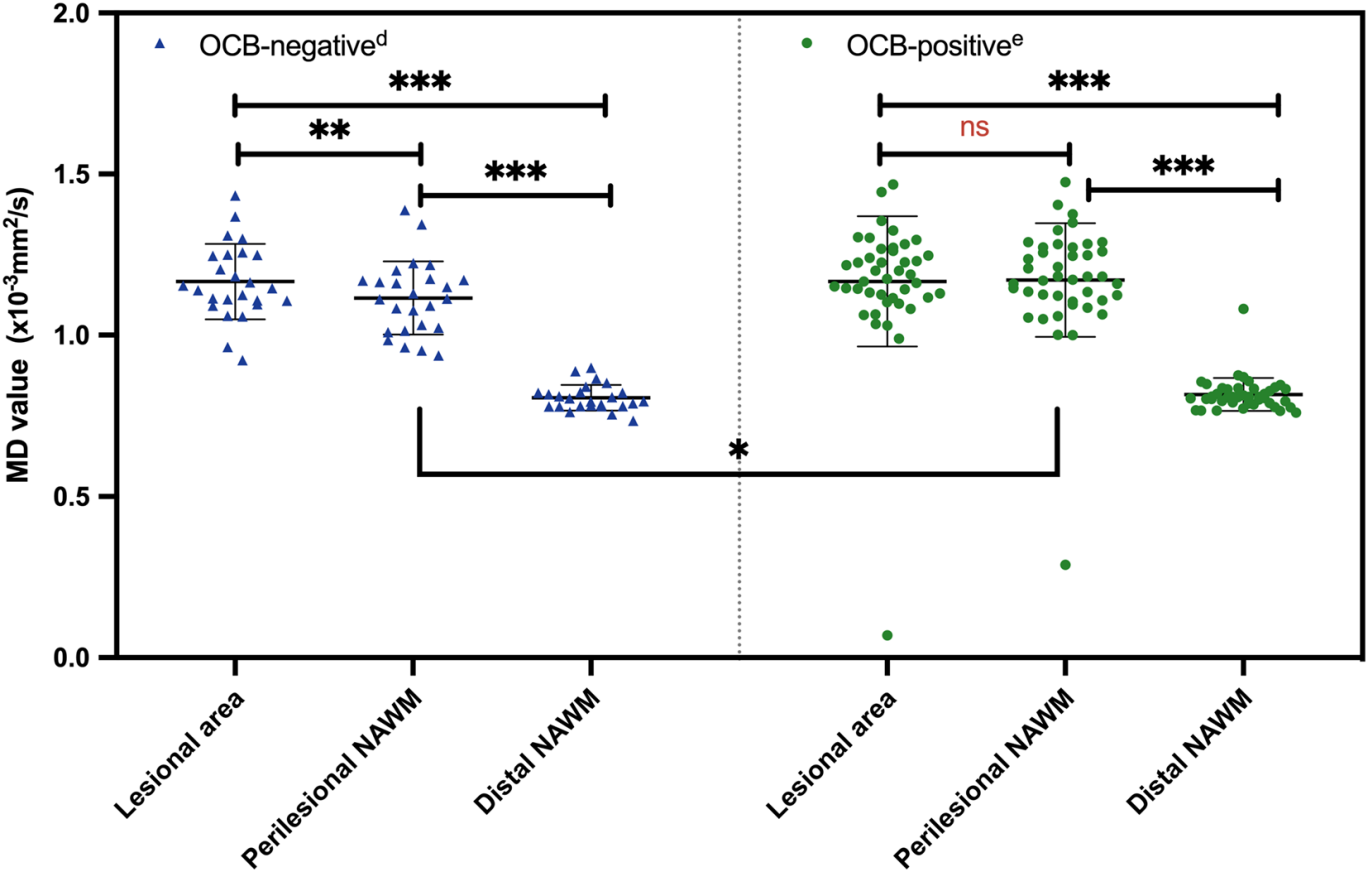
Comparison of mean skeleton MD values in different white matter regions: lesional area, perilesional and distal NAWM. *OCB* oligoclonal IgG bands, *MD* mean diffusivity, *NAWM* normal-appearing white matter. The horizontal lines were at mean with standard deviation. The difference of the means or ranks was calculated using ^d^paired t-test or ^e^Wilcoxon Signed Ranks test (**p < 0.01, ***p < 0.001). The p values were corrected by Bonferroni method.

The results of lesion distribution and regional brain volumes are shown in Table 2. Cerebellar lesions were more commonly found in the OCB-positive group, which demonstrated a trend towards significance (24% vs. 3%, p = 0.023 and 0.057 before and after adjusting for disease duration). Amygdala volumes were lower in the OCB-positive than OCB-negative group (2.20 vs 2.60 mL, *p* = 0.034) (Fig. 1); while the rest of the baseline regional brain volumes, including the subcortical deep gray matter (SDGM) structures and corpus callosum were not significantly different between groups. For DTI measures (Table 3), we investigated the MD and FA values within 4 regions: (1) lesional area, (2) perilesional normal appearing white matter (NAWM), (3) distal NAWM and (4) corpus callosum. We only found significantly higher MD values in perilesional NAWM in the OCB-positive group (1.173 vs. 1.113 × 10^–3^ mm^2^/s, *p* = 0.043); other DTI measures in other regions were comparable between groups. TBSS also revealed no significant findings in terms of mean skeleton FA and MD value (Fig. 2). In both groups, there was a gradient change in the MD values from the lesional area to the perilesional NAWM to the distal NAWM, with the exception of similar MD values between lesional area and perilesional NAWM in the OCB-positive group (*p* = 0.464 after Bonferroni correction) (Fig. 3). There was no significant correlation between EDSS score and DTI metrics in both OCB-positive group and OCB-negative group.

**Table 2:** Lesion characteristics and brain atrophy measures of all RRMS patients according to CSF-OCB status.

	ALL (n = 75)	OCB-negative (n = 29)	OCB-positive (n = 46)	Adjusted *p*-value
**Lesion characteristics**				
Distribution, n (%)				
Periventricular lesions	72 (96.0%)	27 (93.1%)	45 (97.8%)	0.570^a^
Corpus callosal lesions	53 (70.7%)	20 (69.0%)	33 (71.7%)	0.606^a^
Juxtacortical lesions	45 (60.0%)	15 (51.7%)	30 (65.2%)	0.471^a^
Brainstem lesions	21 (28.0%)	7 (24.1%)	14 (30.4%)	0.536^a^
Cerebellar lesions	12 (16.0%)	1 (3.4%)	11 (23.9%)	0.057^a^
Temporal lobe lesions	54 (72.0%)	19 (65.5%)	35 (76.1%)	0.299^a^
Spinal cord lesions	30 (42.3%)	12 (42.9%)	18 (41.9%)	0.592^a^
Total volume, mL, adjusted mean (SD)	11.38 (11.89)	10.48 (11.07)	11.95 (12.46)	0.737^b^
**Normalized brain volumes, mL, adjusted mean (SD)**				
White matter	742.26 (372.78)	744.52 (343.02)	740.83 (389.10)	0.669^b^
Gray matter	679.53 (462.51)	684.62 (568.69)	676.32 (379.80)	0.459^b^
Peripheral gray matter	583.04 (323.71)	584.16 (300.64)	582.34 (339.00)	0.816^b^
Whole brain	1,421.78 (703.77)	1,429.13 (715.83)	1,417.15 (689.60)	0.645^b^
Thalamus	17.41 (2.54)	17.62 (2.60)	17.27 (2.48)	0.743^b^
Caudate	9.15 (1.27)	9.14 (1.36)	9.15 (1.21)	0.999^b^
Putamen	12.00 (2.00)	12.13 (2.14)	11.92 (1.90)	0.33^b^
Globus pallidus	4.34 (0.83)	4.52 (0.83)	4.22 (0.80)	0.450^b^
Hippocampus	9.74 (1.48)	9.75 (1.50)	9.73 (1.48)	0.968^b^
Amygdala	2.35 (0.73)	2.60 (0.76)	2.20 (0.66)	**0.034**^**b**^
Accumbens	1.18 (0.28)	1.17 (0.26)	1.18 (0.29)	0.834^b^
Corpus callosum	42.91 (5.00)	42.79 (5.12)	42.99 (4.98)	0.865^b^

All *p*-values were adjusted for multiple comparisons using the false discovery rate method.

Significant *p*-values are shown in bold.

*RRMS* relapsing–remitting multiple sclerosis, *CSF* cerebrospinal fluid, *OCB* oligoclonal IgG bands, *SD* standard deviation.

The difference of the means was calculated using ^a^Pearson’s chi-square test or ^b^ANCOVA, and adjusted for disease duration.

**Table 3:** Baseline DTI measures, reported as mean (standard deviation), for all RRMS patients according to CSF-OCB status.

	ALL (n = 67)	OCB-negative (n = 25)	OCB-positive (n = 42)	Adjusted *p*-value
**Lesional area**				
FA	0.317 (0.056)	0.320 (0.406)	0.315 (0.063)	0.989
MD (× 10^−3^ mm^2^/s)	1.167 (0.202)	1.166 (0.117)	1.167 (0.202)	0.568
**Perilesional NAWM**				
FA	0.372 (0.032)	0.376 (0.035)	0.370 (0.030)	0.383
MD (× 10^−3^ mm^2^/s)	1.150 (0.157)	1.113 (0.114)	1.173 (0.176)	**0.043**
**Distal NAWM**				
FA	0.432 (0.024)	0.434 (0.029)	0.431 (0.021)	0.834
MD (× 10^−3^ mm^2^/s)	0.801 (0.110)	0.805 (0.040)	0.798 (0.136)	0.430
**Corpus callosum**				
FA	0.474 (0.082)	0.468 (0.117)	0.478 (0.053)	0.775
MD (× 10^−3^ mm^2^/s)	1.081 (0.140)	1.054 (0.135)	1.097 (0.152)	0.390

The difference of the means was calculated using ANCOVA, and adjusted for disease duration.

All *p*-values were adjusted for multiple comparisons using the false discovery rate method.

Significant *p*-values are shown in bold.

*CSF* cerebrospinal fluid, *OCB* oligoclonal IgG bands, *FA* fractional anisotropy, *MD* mean diffusivity, *NAWM* normal appearing white matter.

### Brain volume loss (BVL) rate

**Figure 4:**
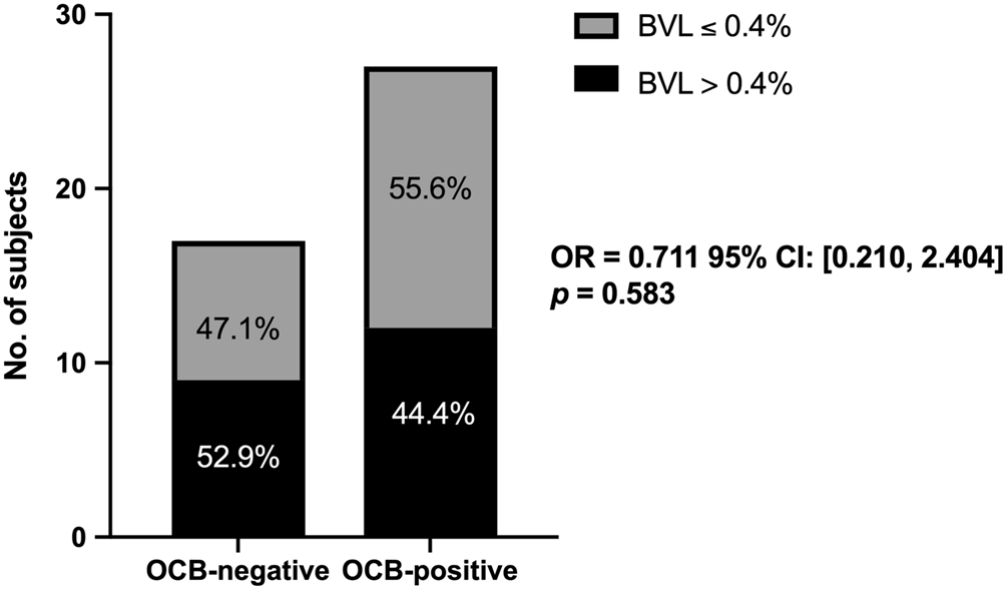
Comparison of brain atrophy rates of OCB-negative and OCB-positive groups in 44 patients with follow-up MRI at ~ 1 year. *OCB* oligoclonal IgG bands, *BVL* brain volume loss, *OR* odds ratio, *CI* confidence intervals. Pearson’s chi-square test was used to compare the difference of brain atrophy rate between the two groups.

Forty-four patients had follow-up brain scans with an interval of 1.25 ± 0.5 years. Given the variability in interval scan times, we adjusted the BVL rate by the follow-up scan interval period. In this subpopulation, the OCB-positive and OCB-negative groups were not significant different in terms of demographics, clinical and imaging features (Supplementary Table). Using the annual BVL threshold of 0.4%, we found no association between CSF-OCB status and BVL rate (Fig. 4).

## Discussion

To our knowledge, this is the first longitudinal study investigating advanced MRI characteristics and CSF-OCB status in a cohort of Chinese RRMS patients. We combined structural and diffusion MRI imaging substrates and evaluated the role of CSF-OCB in relation to disease progression. Our results show that OCB-positive patients had lower amygdala volumes and greater microstructural damage in perilesional NAWM. Otherwise we found that the OCB-positive group shared similar MRI characteristics with the OCB-negative group, including BVL rate.

In our study, Hong Kong Chinese with RRMS showed a 61% OCB-positive rate. This is comparable with reported rates in southern Chinese and Japanese populations, but is lower than Caucasians^15,16^. Nevertheless, more recent studies have identified similar rates (61–62%) of OCB-positive MS patients in Australia and Spain^17,18^. The variation in seroprevalence of CSF-OCB could be related to different study designs and studied population^19^. In clinical aspects, OCB-positive and OCB-negative groups were similar, which is in line with most previous studies^20^.

In terms of lesion characteristics, we only observed a trend for higher proportion of cerebellar lesions in the OCB-positive group. Otherwise, both groups were comparable in terms of lesion distribution and lesion load. Our results agree with previous studies that have shown higher infratentorial lesion load in OCB-positive subjects and supports a trend that OCB-positivity doubles the risk for increased infratentorial involvement^21,22^. These findings may bolster the association between CSF-OCB status and disease pathogenesis; however, this will require further elucidation since most focal white matter lesions in MS are pathological consequences of inflammatory activity. Our study was unable to replicate the increased corpus callosal and juxtacortical lesions, and larger periventricular lesions in OCB-positive MS patients that have been reported in other studies^22,23^.

BVL is a well-established MRI measure in MS patients, and is a promising biomarker for predicting disability progression. Globally, gray matter (GM) atrophy drives disability progression^24^. Regionally, macro- and microdamage of substructures such as the thalamus and corpus callosum are related to physical and cognitive deficits in MS^8,25^. Atrophy of the caudate nucleus, putamen, and globus pallidum have also been implicated, though with more inconsistent results^8,26^. We found only one cross-sectional study that investigated the relationship between CSF-OCB status and regional brain volumes. A study from the Swedish MS registry which recruited 28 OCB-negative and 35 age- and sex-matched OCB-positive RRMS and SPMS patients reported larger white matter (WM) lesion volumes, smaller hippocampal GM volume, and smaller corpus callosal and brainstem WM volumes in the OCB-positive group^27^. In contrast, our study on Chinese RRMS patients only showed significantly reduced amygdala GM volume in the OCB-positive group, whilst other regional brain volumes were similar. In this case, direct comparison of both studies may not be applicable, given the different disease phenotypes studied and possible effect of ethnicities. Meanwhile, the role of the amygdala in MS is not clear. Some association with fatigue has been proposed^28,29^ and could spur further investigations on the relationship between CSF-OCB status and fatigue.

In this study we used DTI to investigate the microstructural changes in white matter. Increased MD and decreased FA values reflect axonal damage and have been consistently demonstrated in lesional areas and NAWM of MS patients^10^. In line with other studies^30^, we observed a gradient of MD from lesional area to perilesional NAWM to distal NAWM. Interestingly, the gradient was partly disrupted in the OCB-positive group, where similar MD values between perilesional NAWM and lesional area were instead observed. Further, the perilesional NAWM MD of the OCB-positive group was significantly higher than the OCB-negative group, suggesting greater microstructural damage. To our knowledge there are no other DTI studies in MS patients investigating the same white matter regions in relation to CSF-OCB status. Whether association between greater microstructural injury in the perilesional NAWM and OCB-positivity will require confirmation with a larger sample size. Further, longitudinal assessment of lesion growth, among other MRI variables, could be an additional avenue of research for understanding the clinical implications of perilesional NAWM changes in relation to CSF-OCB status. Meanwhile microstructural damage in the corpus callosum, which relates to physical and cognitive disability^12^, did not differ between the OCB-positive and OCB-negative groups. With regard to disability, the DTI metrics did not correlate with EDSS scores. Alternative measures such as neuropsychological assessment of cognitive functions may be more sensitive to detect clinical manifestations of microstructural injury.

The presence of CSF-OCB in CIS is an independent risk factor for developing clinically definite MS^31^. However, whether CSF-OCB status could inform prognosis in MS remains controversial^3,5^. In our study, It appears unlikely that CSF-OCB played a significant role in predicting disease progression and prognosis from both clinical and radiological perspectives: (1) the EDSS scores as well as EDSS progression did not differ significantly between the two groups; (2) only one SDGM structure (amygdala) showed significant difference between the two groups, the implication of which is unknown; (3) microstructural metrics that may contribute to disability did not differ between the two groups. Further studies with larger sample size and longer follow-up period are needed to determine the role of CSF-OCB in predicting prognosis and therapy response.

Our work is not without limitations. First, studies with larger sample size in Chinese population are warranted to expand the applicability of our results. Second, we did not assess cortical lesion load, which have been reported to be increased in OCB-positive patients and related to disability progression^32^. Third, although EDSS is the most commonly used clinical tool for evaluating MS, it is heavily biased to gait assessment. Evaluation of severity of cognitive impairment may be more sensitive for correlational analysis; neuropsychological testing should be incorporated to complement EDSS assessments^32,33^. Fourth, our study included subjects with relatively short follow-up time. Further studies with longer follow-up duration are needed to confirm whether brain atrophy rate differ between OCB-negative and OCB-positive groups.

## Methods

### Participants

We recruited RRMS patients from the prospective Chinese University of Hong Kong—Multiple Sclerosis Registry (CUMSR). All patients were diagnosed or verified using the 2017 McDonald criteria and prospectively followed up in the MS clinic^1^. The patients included were relapse-free for at least 3 months and tested for CSF-OCB status. We excluded patients: (1) with history of other neurological diseases; (2) who have received DMTs for less than 6 months to avoid probable pseudoatrophy in brain volumetric analysis^34^. Demographics, clinical variables including sex, age at disease onset, disease duration, ARR, EDSS scores, DMTs used, laboratory and MRI data were collected. Baseline ARR was calculated by dividing the total number of clinical relapses by disease duration (years) from disease onset to entry visit. EDSS scores were assessed along with MRI within 24 h; The EDSS progression was defined as any 0.5-point sustained (3-month) increase in the EDSS score in patients who had a baseline EDSS score of ≥ 6.0 or 1.0-point sustained (3-month) increase in the EDSS score in patients who had a baseline EDSS score of ≥ 1.0 or any ≥ 1.5-point sustained (3-month) increase in the EDSS score in patients who had a baseline EDSS score of 0.0. All participants provided written informed consent. The study was approved by Joint Chinese University of Hong Kong—New Territories East Cluster Clinical Research Ethics Committee (CRE-2013.155 & CRE-2014.130). All methods were carried out in accordance with relevant guidelines and regulations.

### CSF-OCB detection

The OCB detection was carried out by isoelectric focusing (IEF) and lgG-specific immunofixation^35^. CSF-OCB positivity was defined as two or more bands present in CSF but absent in plasma at the same point. If CSF-OCB were present on at least one examination, the patient was classified as OCB-positive^36^.

### MRI acquisition and analysis

All MRI scans were performed on a single 3 T scanner (Philips Achieva TX, Best, The Netherlands) using an 8-channel head coil. Structural imaging included volumetric T1-weighted and FLAIR sequences of the brain and T2-weighted sequences of the spinal cord which were all acquired in the sagittal plane. Their parameters were as follows (TR: repetition time, TE: echo time, TI: inversion time, FOV: field of view): (1) 3D T1-weighted Fast Field Echo (FFE): TR 7.5 ms, TE 3.5 ms, FOV 250 mm, matrix 228 × 208, slice thickness 1.1 mm; (2) 3D T2-weighted FLAIR with fat suppression: TR 8,000 ms/TI 2,400 ms, TE 341 ms, FOV 230 mm, matrix 208 × 208, slice thickness 1.1 mm; (3) T2-weighted: TR 3,666 ms, TE 120 ms, FOV 270 mm, matrix 340 × 254, slice thickness 2 mm.

DTI of the whole-brain was acquired using a single shot echo-planar imaging diffusion-weighted sequence (TR = 9,060 ms, TE = 60 ms, FOV = 224 mm, matrix 112 × 112, 70 axial slices with an isotropic 2 mm resolution) with 32 gradient directions with non-collinear diffusion gradients (b-value of 1,000 s/mm^2^) and one volume without diffusion weighting (b-value of 0 s/mm^2^).

### Image processing and analysis

#### Lesion Distribution and volume

White matter lesion (WML) segmentation was performed by lesion growth algorithm (LGA) in lesion segmentation tool (LST), which is an open source toolbox for Statistical Parametric Mapping (SPM) 12.0^37^. LGA requires a T1-weighted image in addition to the FLAIR image. After fully automatic processing by LST, the lesion mask and volumetric result for each subject was obtained. Lesions were filled on 3D T1-weighted FFE images by lesion filling tool in LST^38^. An experienced neurologist visually checked the native brain FLAIR and spinal cord MRI images and recorded the areas of distribution of lesions.

#### Global and regional brain atrophy measures

For baseline analysis, SIENAX software from the FMRIB Software Library (FSL) v 6.0 (www.fmrib.ox.ac.uk/fsl/) was used. Lesion map was filled by LST. Normalized whole-brain, normalized gray matter (GM), normalized white matter (WM) volume and normalized deep gray matter were measured as previously described^39^. Absolute tissue volumes for the thalamus, caudate, putamen, globus pallidus, hippocampus, amygdala, and nucleus accumbens at baseline were estimated from lesion-filled 3D T1-weighted images with FMRIB’s Integrated Registration and Segmentation Tool (FIRST), a model-based segmentation/registration tool^40^. Normalized subcortical deep gray matter (SDGM) volumes were obtained by multiplying the estimated volumes from this tool by the volumetric scaling factor from SIENAX. For corpus callosal volume, the mask in the Montreal Neurological Institute (MNI152) 2 mm standard space was generated by using edit mode in FSLeyes. The brain was extracted from 3D T1-weighted images using BET in FSL^41^. The skull-removed T1-weighted images were registered to MNI152 2 mm standard space images with 12 DOF affine registration implemented in FLIRT and were refined by non-linear registration implemented in FNIRT in FSL v6.0^42,43^. Secondly, the corpus callosum mask was inverted to native T1 space by applying transformation matrices and warp fields. A threshold of 0.5 was applied for native space masks. The ‘fslstats’ command was then used to extract volumetric data on white matter segmentation image output from SIENAX. For longitudinal changes of the whole brain volume, we applied the SIENA method to calculate the percentage of brain volume change (PBVC)^44^.

#### Diffusing tensor image measures and TBSS

All DTI images were preprocessed with Eddy in FSL to correct for distortions due to the applied gradient directions^45^. Subsequently, DTIFIT was used to fit a diffusion tensor model at each voxel and generate individual FA and MD images. FA maps were fed into the Tract-Based Spatial Statistics (TBSS) tool^46^. The FA maps of all subjects were aligned into a 1 × 1 × 1mm standard space called FMRIB58_FA by non-linear registration and averaged to obtain a mean FA skeleton. Finally, each subject’s aligned FA data were projected onto this skeleton. Similar processes were applied to MD maps using the individual registration and projection vectors obtained in the FA nonlinear registration and skeletonization stages. A voxel-wise cross-subject statistics analysis was then performed to compare DTI metrics of OCB-positive and OCB-negative patients. General linear model (GLM) was used to perform group comparison, adjusted for subject’s age and sex, and applied to the spatial maps using permutation-based non-parametric testing (5,000 permutations) with correction for multiple comparisons, by using a cluster-based correction approach and FWE-corrected p-value < 0.05. Relevant WM tracts were localized by using FSL WM Atlas. FA and MD values in various regions of interest (ROI’s) were extracted and computed from their respective maps. The ROI’s included: (1) lesional area, (2) NAWM in perilesional area, defined as 3-voxels around the focal lesion (‘perilesional NAWM’), (3) the rest of NAWM, defined as more than 3-voxel away from the focal lesion (‘distal NAWM’), and (4) corpus callosum.

### Statistical analysis

SPSS version 23.0 was used in the statistical analysis. We used Q-Q plots, Kolmogorov–Smirnov test and Shapiro–Wilk test to assess for normality of data. WML volumes and DTI parameters (with exception of perilesional NAWM MD values and CC FA values) were log transformed due to their skewed distribution. All values were reported as mean (standard deviation) or median (interquartile range) as appropriate. Comparison of demographics and clinical features between groups were carried out by t-test, Mann–Whitney U-tests, and Pearson’s chi-square test as appropriate. Brain volumes and DTI measures between groups were compared using analysis of covariance (ANCOVA) and Pearson’s chi-square test, with adjustment for disease duration. A p-value of < 0.05 was considered as statistically significant. All results were adjusted for multiple comparisons using the false discovery rate (FDR) approach in order to avoid inflating Type I error. We used paired t-test and Wilcoxon Signed Rank tests to compare MD values between different ROI pairs due to their underlying dependency; the results were corrected by Bonferroni method.

## Conclusion

Our study showed that the presence of CSF-OCB was largely uncorrelated with cross-sectional MRI characteristics and brain volume loss over one year. Our findings weaken the role of CSF-OCB in adding additional information to quantitative MRI metrics in relation to disease progression over the observed period, and support the application of quantitative MRI as a more sensitive biomarker for MS.

## Supplementary information

Supplementary Information
