# Supplementary material for: Advanced MRI features in relapsing multiple sclerosis patients with and without CSF oligoclonal IgG bands
Source: Sci Rep. 2020 Aug 13;10:13703. doi: 10.1038/s41598-020-70693-9 (PMC7426866; doi:10.1038/s41598-020-70693-9)
Supplement: Supplementary file 1 — Supplementary Information [file 41598_2020_70693_MOESM1_ESM.pdf]

Advanced MRI Features in Relapsing Multiple Sclerosis Patients with and without CSF Oligoclonal IgG Bands

Authors

Lin Zhao[1], Jill Abrigo[2], Qianyun Chen[2], Cheryl Au[1], Angel Ng[1], Ping Fan[3], Vincent Mok[1], Wei Qiu[3], Allan G. Kermode[4], Alexander Y. Lau[1] *

Affiliation

[1] Department of Medicine and Therapeutics, Prince of Wales Hospital, Chinese University of Hong Kong, Hong Kong SAR, China
[2] Department of Imaging and Interventional Radiology, Prince of Wales Hospital, Chinese University of Hong Kong, Hong Kong SAR, China
[3] Neurology Department, Third Affiliated Hospital, Sun Yat-sen University, Guangzhou, China
[4] Centre for Neuromuscular and Neurological Disorders, Perron Institute, University of Western Australia, Perth, Australia

Corresponding Author
Dr. Alexander Y. Lau
Department of Medicine and Therapeutics, Prince of Wales Hospital, Chinese University of Hong Kong, Hong Kong SAR, China
Tel: +852 28733097; Fax: +852 26493761
Email: alexlau@cuhk.edu.hk

Keywords
Multiple sclerosis, Oligoclonal bands, MRI, Chinese

Supplementary table. Baseline demographics, clinical characteristics and annual brain atrophy rate for patients with follow-up MRI data, based on the CSF-OCB status.

	**ALL (n=44)**	**OCB-negative (n=17)**	**OCB-positive (n=27)**	**p value**
Female, sex, n (%)	34 (77.3%)	14 (82.4%)	20 (74.1%)	0.716[a]
Age at disease onset, years, mean (SD)	28.64 (8.30)	30.21 (9.33)	27.67 (7.60)	0.326[b]
Disease duration, years median (IQR)	4.41 (1.58-7.58)	3.78 (1.25-5.33)	5.0 (2.14-9.76)	0.123[c]
ARR before baseline median (IQR)	0.31 (0.10-0.79)	0.41 (0.09-0.82)	0.3 (0.08-0.78)	0.698[c]
EDSS progression, yes, n (%)	4 (9.1%)	2 (11.8%)	2 (7.4%)	0.634[a]
DMT application during FU, n (%)	36 (81.8%)	12 (70.6%)	24 (88.9%)	0.227[a]
FU duration, months median (IQR)	1.14 (0.95-1.51)	1.09 (0.90-1.38)	1.19 (0.96-1.61)	0.433[c]
BVL rate (%), median (IQR)	-0.32 (-0.73-0.14)	-0.46 (-0.71-0.12)	-0.26 (-1.16-0.17)	0.933[c]

CSF: cerebrospinal fluid; OCB: oligoclonal bands; ARR: annual relapse rate; DMT: disease modifying therapy; FU: follow-up; BVL: brain volume loss; SD: standard deviation; IQR: interquartile range

The difference of the means or ranks was calculated using [a] Pearson chi-square test [b] the Student t test or [c] Mann-Whitney rank sum test
